# Protective Effects of Polysaccharides from Soybean Meal Against X-ray Radiation Induced Damage in Mouse Spleen Lymphocytes

**DOI:** 10.3390/ijms12118096

**Published:** 2011-11-17

**Authors:** Lei Yao, Zhenyu Wang, Haitian Zhao, Cuilin Cheng, Xiaoyi Fu, Jiaren Liu, Xin Yang

**Affiliations:** 1 School of Food Science and Engineering, Harbin Institute of Technology, 73 HuangHe Road, NanGang District, Harbin 150090, China; E-Mails: yaoleiyl2000@163.com (L.Y.) zhaoht9999@163.com (H.Z.); ccuilin@hit.edu.cn (C.C.); yangxin940@163.com (X.Y.); 2 National Research Center of Soybean Engineering and Technology, Northeast Agriculture University, 201 GongBin Road, XiangFang District, Harbin 150030, China; E-Mail: fuxiaoyi209@163.com; 3 School of Forestry, Northeast Forestry University, 26 HeXing Road, DongLi District, Harbin 150040, China; 4 Harvard Medical School, 300 Longwood Avenue, Boston, MA 02115-5737, USA; E-Mail: Jia-ren.liu@childrens.harvard.edu

**Keywords:** soybean meal polysaccharides, spleen lymphocytes, radioprotective, comet assay

## Abstract

The aim of this study was to investigate radioprotective effect of the polysaccharides from soybean meal (SMP) against X-ray radiation-induced damage in mouse spleen lymphocytes. MTT and comet assay were performed to evaluate SMP’s ability to prevent cell death and DNA damage induced by radiation. The results show that, X-ray radiation (30 KV, 10 mA, 8 min (4 Gy)) can significantly increase cell death and DNA fragmentation of mouse spleen lymphocytes. Pretreatment with SMP for 2 h before radiation could increase cell viability, moreover, the SMP can reduce X-ray radiation-induced DNA damage. The percentage of tail DNA and the tail moment of the SMP groups were significantly lower than those of the radiation alone group (*p* < 0.05). These results suggest SMP may be a good candidate as a radioprotective agent.

## 1. Introduction

Ionizing radiation causes a variety of biological effects in living cells. DNA is one of the key targets for ionizing radiation induced damage in most living organisms ranging from bacteria to humans. Exposure to ionizing radiation inflicts single strand breaks, double strand breaks, base damage and DNA-protein cross-links in the genomic DNA [1]. These lesions in DNA, if misrepaired or unrepaired, may lead to mutations and cancer in mammals [2].

Therefore, search for the chemical or biological agents that are able to protect human beings from the ionizing radiation is a key issue in radiation biology. Numerous drugs of both synthetic and natural origin, e.g., antioxidants [3,4], sulfhydryl compound, estrogens, haemopoietic growth factors and cytokines [5] have been tested in both *in vitro* and *in vivo* models to mitigate injuries caused by ionizing radiation. Amifostine (Ethyol^®^) is the only radio-protector that has been approved by the Food and Drug Administration (FDA), USA [6]. However, the radioprotective effects of phosphorothioate compounds, including amifostine, are short term, and associated with severe side effects (e.g., nausea, vomiting, diarrhoea, hypotension, hypocalcaemia, nephro- and neuro-toxicity) at clinically effective doses [7]. These limitations have greatly restricted their clinical use.

Hence, the search for newer, less toxic and more effective radioprotector sources has been on going for several decades. Much attention has recently been focused on finding potential radioprotectors, especially, from nature plant origins, which are capable of modifying immune and radiation responses without other side effects [8].

Investigations show that polysaccharides have many biological activities, such as antioxidant activity [9,10], antitumor activity [11], antiradiation activity [2,12] and immunomodulatory activity [13]. Soybean meal is a co-product from the processing of soybean oil and soybean protein isolate, its main component is cereal cell wall polysaccharide which has attracted attention with its beneficial effects on human health [14]. However, the cell wall polysaccharide of soybean has very low solubility in water, which restricts its applications. Until now, it is usually used as feed or discarded, which undoubtedly is a waste of resource. In the present study, a water soluble soybean meal polysaccharide (SMP) was prepared by enzymatic hydrolysis of the cell wall polysaccharide of soybean. Radioprotective effect of SMP on mouse spleen lymphocytes exposure to X-ray radiation was investigated by MTT and comet assay.

## 2. Results and Discussion

### 2.1. Effects of SMP on Cell Viability in X-Irradiated Spleen Lymphocytes

**Figure 1:**
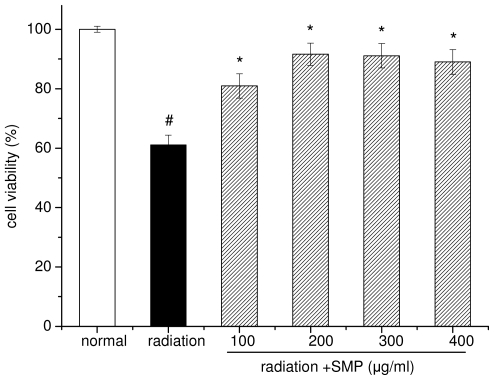
Effect of polysaccharides from soybean meal (SMP) on cell viability of X-ray radiated lymphocytes. (# *p* < 0.05 compared to the value of normal group; * *p* < 0.01 compared to the value with radiation group). Values are given as means ± S.D. of three independent experiments in each group.

The effect of SMP on cell viability in X-ray radiation-induced spleen lymphocytes was measured via MTT assay and results are shown in Figure 1. X-ray radiation treatment significantly inhibited the growth of the mouse spleen lymphocytes. The cell viability of radiation alone group was reduced to 58.12%. Addition of SMP in culture medium can significantly reduce the death of cell-radiated at concentrations of 100–400 μg/mL. At dose 100 μg/mL, the cell viability was 80.95%, under concentration of 200 μg/mL, reach the best protective effect (cell viability 91.62%), when the concentration of SMP reach 400 μg/mL, protective effects no longer increases (cell viability 90.02%).

### 2.2. Effects of SMP on X-ray Radiation-Induced DNA Damage

Ionization radiation induced damage to cellular DNA is mainly due to strand breaks and may lead to loss of viability of cells. The comet assay (single cell gel electrophoresis) is a quick, simple, sensitive and reliable technique used to detect primary DNA damage induced by chemicals and radiation in individual mammalian cells [15]. Exposure of lymphocytes to X-ray radiation resulted in an increase in comet attributes such as tail length (TL), tail moment (TM), olive tail moment (OTM) and % DNA in the tail [16]. In the present study, the comet assay was used to evaluate the DNA damage induced by X-ray-radiation in mouse spleen lymphocytes that had been incubated in the presence or absence of SMP.

**Figure 2:**
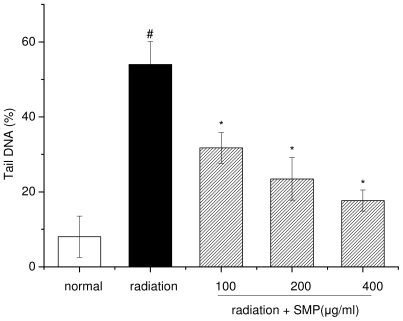
Dose-dependent effect of SMP on percentage DNA in tail induced by 4 Gy X-ray radiation. (# *p* < 0.05 compared to the value of normal group; * *p* < 0.05 compared to the value of radiation group).

**Figure 3:**
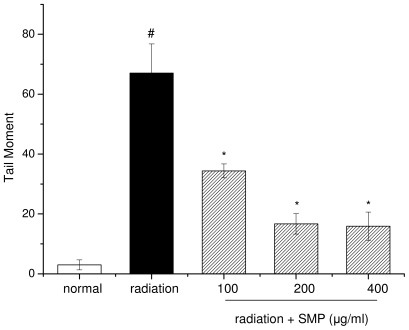
Dose-dependent effect of SMP on Tail Moment induced by 4 Gy X-ray radiation. (# *p* < 0.05 compared to the value of normal group; * *p* < 0.05 compared to the value of radiation group).

According to the results of comet assay, the exposure of mouse spleen lymphocytes to radiation increased comet parameters like tail length, percent of DNA in tail and tail moment suggesting radiation-induced damage to DNA. As seen in Figures 2 and 3, the mean comet tail DNA percentage in the radiation alone group was 53.99 ± 6.09, which was significantly higher than 8.04 ± 5.53 in the normal group (*p* < 0.01). The median value of tail moment (TM) was 67.06 ± 9.77 in the radiation alone group, significantly higher than 3.0 ± 1.67 in the normal group (*p* < 0.01). When compared to the radiation alone group, pretreatment with SMP decreased the comet parameter at all concentrations (100, 200, 400 μg/mL), in a dose-response manner (*p* < 0.05). Among all concentrations, 400 μg/mL concentration of SMP can decrease the % DNA in tail to 17.69 ± 2.82 and tail moment 15.92 ± 4.71. Hence, these results indicate that pre-incubated with SMP can significantly reduce the DNA damage induced by X-ray radiation, and the results are consistent with data of cell viability obtained by MTT assay.

It is well known that, free radical production is also a primary basis for the particular danger to biological systems of ionizing radiation. Radiation generates reactive oxygen species (ROS) such as superoxide anions, hydrogen peroxide, and hydroxyl radicals, which show high reactivity to a variety of cellular macromolecules, including DNA, lipids, and proteins. Therefore, compounds capable of reducing the free radical activity could be useful as radio protector.

Many kinds of polysaccharides such as polysaccharides from soybean [10], *Auricularia auricular* polysaccharide [17], *Lycium barbarum* polysaccharide [18], tea polysaccharide [19], *Longan* polysaccharide [20] and *Litchi chinensis* polysaccharide [21] exhibit antioxidant and free radical-scavenging activities. The antioxidant mechanism may be due to the supply of hydrogen by polysaccharides, which combines with radicals and, itself, forms a stable radical to terminate the radical chain reaction. The other possibility is that polysaccharides can combine with the radical ions that are necessary for radical chain reaction; then the reaction is terminated. Our previous studies have found that SMP have evident scavenging activity against hydroxyl radical and DPPH radical *in vitro* (data not shown). We hypothesized that protection effect of SMP against damage induced by X-ray radiation was partly due to its free radical scavenging ability. However, besides the oxidant injury, ionizing radiation may induce damage in cells or organisms through other pathways, such as attack biomacromolecules (DNA, protein and lipid) directly; further studies are needed to find out the mechanism of radioprotection of SMP. In any case, the study can provide a theoretical and experimental basis for developing natural radioprotector. We have analyzed the monosaccharide composition of SMP by gas chromatography-mass spectrometry (GC-MS) method [22,23]. In comparison to the monosaccharide standards, SMP consists of ribose, rhamnose, arabinose, xylose, mannose, glucose and galactose in a molar ratio of 1:1.19:3.38:0.77:7.81:86.42:21.81. Correlation studies are in progress.

## 3. Experimental Section

### 3.1. Chemicals and Reagents

3-(4,5-dimethylthiazol-2-yl)-2,5-diphenyltetrazolium bromide(MTT), Red Blood Cell (LBC) Lysis Buffer, Tris (hydroxymethyl) aminomethane, dimethylsulfoxide (DMSO) and ethylenediaminetetraacetic acid disodium salt (EDTA-2Na) were purchased from Sigma Chemical Co., USA. Ethidium Bromide (EB), RPMI 1640, fetal calf serum, Triton X-100, *N*-Lauroyl Sarcosine Sodium and other chemicals in the studies were of highest quality commercially available from local suppliers (Sinopharm Chemical Reagent Beijing Co., Ltd, China).

### 3.2. Preparation of Soybean Meal Polysaccharides (SMP)

Soybean meal, after alkali-soaking (3% NaOH, 100 °C, 1 h) and acid-soaking (3% H_2_SO_4_, 100 °C, 1 h) was washed to neutral and then dried at the temperature 50 °C. The pretreated soybean meal was then hydrolysed using a commercial cellulase (Accellerase™ 001) solution supplied by Genencor International. The reaction was conducted in a thermostated stirring reactor for 6 h, under the conditions of liquid-solid ratio of 5% (w/v); initial enzyme concentration was 1% (v/v); pH 5.0; reaction temperature 50 °C (maximum enzymic activity). Hydrolyzed liquid was centrifuged (4000× g, 10 mim) then supernatant was collected and concentrated by rotary evaporator. Four times volumes of 95% of ethanol was then added into the concentrated solution, and centrifuged (4000× g, 10 min) to precipitate polysaccharides, and the procedure was duplicated for two times. Finally, the powder (SMP) was freeze-dried under vacuum for 20 h at −40 °C.

### 3.3. Preparation of Mouse Spleen Lymphocytes

Approximately 7–8 weeks old ICR male mice weighing 20 ± 2 g, were obtained from Harbin Veterinary Research Institute, (CAAS, license number: SCXK(HEI)2006-009). Spleens of mice were chopped in Dulbecco’s phosphate-buffered saline and passed through a stainless steel mesh. The spleen cell suspensions were centrifuged at 120× g for 10 min. Sediment was treated with red blood cell break solution for 3–5 min and washed twice with PBS. The lymphocytes were then obtained by centrifugation, final single cell suspensions (1–1.5 × 10^6^ cells/mL) were prepared in RPMI 1640 medium supplemented with l-glutamine (2 mM), penicillin (100 units/mL), streptomycin (100 μg/mL), and 10% fetal bovine serum.

### 3.4. X-ray Radiation

The cells were pre-treated with different concentration of SMP (0, 100, 200, 400 μg/mL) at 37 °C in a humidified atmosphere of 5% CO_2_ for 2 h and then radiated. X-ray radiation was performed using apparatus (Soft X-ray generator, HY-35, XYTEST Equipment Co., Ltd. Hunan, China) operating at 30 KV, 10 mA at a dose rate of 0.5 Gy/min, 8 min at room temperature. Total absorbed dose was 4 Gy.

### 3.5. Determination of Cell Viability

The effect of SMP on the cell viability was determined using the MTT (3-(4,5-dimethylthiazol-2- yl)-2,5-diphenyltetrazolium bromide) assay [24]. 100 μL lymphocytes pre-treated with different concentration of SMP (0, 100, 200, 400 μg/mL) were seeded in a 96-well plate at a concentration of 5 × 10^4^ cells/mL using RPMI 1640. After 2 h incubation (5% CO_2_, 37 °C), the cells were X-irradiated and then further incubated for 1 h. MTT stock solution (5 mg/mL in PBS) was then added to each well for a total reaction volume of 110 μL. After incubating for 4 h, the supernatants were centrifuged to remove untransformed MTT and 100 μL of DMSO was added to each well to dissolve formazan crystals. The optical density (O.D.) of each sample was measured at 540 nm using an ELISA microplate reader (Bio-Rad 550, Hercules, CA, USA). Cell viability was expressed as the ratio of optical densities of the treatment group to normal (unirradiated) group and was calculated using the equation showed below. Cell viability differences were compared using paired student *t*-test.

$$\text{cell viability }(\%)=\frac{\text{O}.\text{D}.(\text{experiment})-\text{O}.\text{D}.(\text{blank})}{\text{O}.\text{D}.(\text{control})-\text{O}.\text{D}.(\text{blank})}\times 100\%$$

### 3.6. Determination of DNA Damage (Comet Assay)

The comet assay is a single-cell gel electrophoresis technique based on the principal that DNA with nicks releases fragments that migrate farther in an electric field than undamaged DNA. The comet assay was performed according to Ramachandran *et al*., with slight modifications [25]. About 100 μL of 1% normal agarose was layered on each slide, covered with a cover-slip and put at 4 °C for solidification. The same volume of cells were collected and mixed with 1% low melting agarose at 37 °C in a ratio 1:1. From this mixture, 100 μL were laid on top of the first gel and covered with a cover-slip as before. Once the upper layer solidified, the cover-slips were gently removed and slides were carefully immersed in prepared cold lysis solution (2.5 mM NaCl, 100 mM Na_2_EDTA, 10 mM Tris, to mix 1% Triton X-100, 10% DMSO, 1% *N*-Lauroyl Sarcosine Sodium before use) for 1 h at 4 °C in dark place. After slides were removed from the lysis solution and immerged in an electrophoresis buffer (0.3 M NaOH, 1 mM Na_2_EDTA, pH 13) for 20 min to allow the DNA to unwind and then carried out electrophoresis at 20 V, 200 mA for 20 min. After electrophoresis, the slides were washed twice with neutralization buffer (0.4 M Tris, pH 7.5) and stained with 20 μL of 20 μg/mL EB solution for 1 min. Slides gel were washed twice with double-distilled water. Comet images were analyzed on an fluorescent microscope (Axio Imager.A1, Carl Zeiss, Germany) equipped with a 365 nm excitation filter and 435 nm barrier filter at 200× magnification and imaged with a networking digital camera (Computer Optics, Germany). Comets were analyzed using the public domain PC-image analysis program CASP software. One hundred cells per sample were randomly selected, *i.e.*, 50 cells were from each of the two replicate slides. The percentage of tail DNA and tail moment (TM) served as the indicator.

### 3.7. Statistical Analysis

All the experiments were performed in triplicate. The results were expressed as mean ± standard deviation and analyzed by SPSS 17.0 for Windows software package. The difference was considered significant at *p* < 0.05. All experiments were performed in triplicate, and results were expressed as mean ± SD.

## 4. Conclusions

The present study is the first to demonstrate the ability of SMP to decrease X-ray-radiation-induced cellular damage in mouse spleen lymphocytes *in vitro*. Our results demonstrate that the polysaccharide from soybean meal (SMP) has protective effect on damage of mouse spleen lymphocytes induced by X-ray radiation. The protective mechanisms of SMP may be attributed to its free radical scavenging activity and protective effect on DNA damage. On the basis of the results obtained, SMP can be used for a variety of beneficial chemo-preventive effects. Further studies on the specific components of SMP and *in vivo* studies are in progress to understand the detailed mechanism by which SMP exerts its radioprotective effect.
